# Immunohistochemical Expression of Novel Therapeutic Targets in Squamous Cell Carcinoma of the Bladder

**DOI:** 10.32604/or.2026.078954

**Published:** 2026-07-16

**Authors:** Lisa J. Frey, Nina Lache, Nikita D. Fischer, Niklas Rölz, Lisa Frey, Maximilian Haack, Gregor Duwe, Stefan Porubsky, Axel Haferkamp, Daniel-C. Wagner, Maximilian P. Brandt

**Affiliations:** 1 Department of Urology and Pediatric Urology, Mainz University Medical Center, Langenbeckstraße 1, Mainz, Germany; 2 Institute of Pathology, Mainz University Medical Center, Langenbeckstraße 1, Mainz, Germany; 3 Translational Oncology (TRON gGmbH), Mainz University Medical Center, Mainz, Germany

**Keywords:** Bladder cancer, NECTIN cell adhesion molecule 4 (NECTIN4), squamous cell carcinoma of the bladder, therapeutic targets

## Abstract

Objectives: Squamous cell carcinoma (SCC) of the bladder is an aggressive histologic subtype with distinct clinical behavior and limited treatment options after platinum-based chemotherapy. This study aimed to evaluate potential therapeutic targets in bladder SCC. Methods: A retrospective cohort of 790 patients who underwent radical cystectomy for bladder cancer between 2011 and 2021 was screened to identify cases with histologically confirmed SCC. All SCC cases in the pathology department from 2003 to 2011 were also reviewed. Clinical and pathological data from 54 patients were analyzed. A tissue microarray (TMA) was constructed, and immunohistochemical (IHC) analyses were performed for programmed death-ligand 1 (PD-L1), nectin cell adhesion molecule 4 (NECTIN4), trophoblast cell-surface antigen 2 (TROP2), human epidermal growth factor receptor 2 (HER2), carcinoembryonic antigen-related cell adhesion molecule 5 (CEACAM5), and CD8^+^ T cells. Expression levels were assessed for prognostic relevance using the log-rank test and Kaplan-Meier survival analysis. Results: A TMA comprising samples from 42 of 54 patients (22 pure SCC, 20 partial SCC) was successfully constructed. NECTIN4 (positive vs. negative) expression, PD-L1 (combined positive score ≥ 10 vs. <10) expression, and CD8^+^ density (high vs. low) showed a nearly equal distribution across the cohort. HER2 expression was detected in 14.3% of cases, CEACAM5 in 21.4%, and TROP2 in 83.3% of tumors. High NECTIN4 expression, increased CD8^+^ density, and administration of adjuvant chemotherapy were associated with improved overall survival. Conclusion: Several actionable targets were identified in bladder SCC, supporting further exploration of targeted therapies.

## 1 Introduction

Squamous cell carcinoma (SCC) of the bladder is an aggressive histologic subtype of bladder cancer (BC), accounting for approximately 5% of cases diagnosed via transurethral resection of the bladder (TURB) [1]. Compared with urothelial carcinoma (UC), SCC is associated with significantly worse overall survival (OS) outcomes [2]. For resectable muscle-invasive SCC, radical cystectomy (RC) remains the gold-standard treatment of choice. Current guidelines recommend immediate surgery without neoadjuvant chemotherapy, as no survival benefit has been shown for this histologic subtype [3]. For SCC after RC, evidence on adjuvant treatment is scarce, and routine use of chemotherapy or immunotherapy is not supported by the limited, mainly platinum-based data available [4]. In the metastatic setting, evidence on the efficacy of targeted or immunotherapeutic agents in SCC is lacking, although recent reports indicate promising activity of the antibody–drug conjugate (ADC) enfortumab vedotin [5,6]. In recent years, novel systemic treatment options, including immune checkpoint inhibitors (ICIs) and ADCs, have emerged for patients with unresectable, locally advanced, or metastatic UC. Due to the absence of SCC-specific treatment guidelines, the management of SCC generally follows standard-of-care regimes established for UC, with consideration of individual patient factors [7]. Notably, SCC remains underrepresented in the pivotal clinical trials that led to the approval of ICIs and ADCs, and is rarely included as a stratification factor in trial design [8]. In contrast to UC, where FGFR3 mutation testing enables targeted therapies, such approaches are rarely employed in SCC patients [9,10].

Several biomarkers, including programmed cell death protein 1 (PD-1), programmed death-ligand 1 (PD-L1), nectin cell adhesion molecule 4 (NECTIN4), trophoblast cell surface antigen 2 (TROP2), and human epidermal growth factor receptor 2 (HER2), have been identified as potential targets for ICI and ADC-based therapies. Data on the expression of these targets in bladder SCC remain limited, often derived from small patient cohorts in which only a subset of clinically relevant markers has been assessed [11].

The present study aims to investigate the immunohistochemical (IHC) expression patterns and prognostic relevance of PD-L1, NECTIN4, TROP2, HER2, carcinoembryonic antigen-related cell adhesion molecule 5 (CEACAM5), and CD8^+^ T cells in pure and partially squamous differentiated bladder carcinomas.

## 2 Materials and Methods

### 2.1 Data and Patient Selection

A single-center, consecutive database comprising clinical data of all patients who underwent RC for SCC between 2011 and 2021 was established and maintained. Additionally, cases from 2003 to 2011 that were only documented in the pathology department were retrieved. The present study was approved by the local ethics committee of the Rhineland-Palatinate Medical Association (#2024-17564) and was conducted in accordance with the Declaration of Helsinki and the International Ethical Guidelines for Biomedical Research Involving Human Subjects. As this is a retrospective study, no individual informed consent was obtained, and data collection was performed in accordance with German hospital law [§ 136], which permits the use of patient records for scientific research.

### 2.2 Immunohistochemical Staining and Tissue Microarray

A tissue microarray (TMA) was constructed from all formalin-fixed, paraffin-embedded tissue samples, as previously described, with each case represented in triplicate (core diameter 1.5 mm) [12]. Tumor areas, or squamous-differentiated areas in cases with partial squamous differentiation, were annotated by a board-certified pathologist, and TMA cores were randomly selected from these annotated regions.

Immunohistochemical staining was performed to assess the expression of PD-L1, NECTIN4, TROP2, HER2, CEACAM5, and CD8^+^ T cells. PD-L1 and HER2 immunostaining were carried out on the Dako Omnis platform (Agilent Technologies, Santa Clara, CA, USA) using the CE-IVD assays PD-L1 IHC 22C3 pharmDx assay (Agilent Technologies, GE006) and the HercepTest (Agilent Technologies, GE001), respectively, according to the manufacturers’ instructions, without protocol modifications. NECTIN4 (Abcam, ab192033, Cambridge, UK), TROP2 (Abcam, ab214488), CEACAM5 (Ci-P83-1, Santa Cruz Biotechnology, sc-23928, Dallas, TX, USA), and CD8 (Agilent Technologies, M7103) immunostaining was performed using a Thermo Fisher Scientific autostainer (Autostainer 480S, Thermo Fisher Scientific, Waltham, MA, USA). For these stains, EDTA-based antigen retrieval was used, followed by incubation (RT) with NECTIN4 1:1000/30 min with protein block, TROP2 1:2000/30 min, CEACAM5 1:100/20 min, or CD8 ready-to-use/20 min. Visualization was performed using EnVision FLEX with Mouse Linker (Agilent Technologies, K8002) or Rabbit Linker (Agilent Technologies, K8019), as appropriate. Slides were digitized at 40× magnification (Nanozoomer XP, Hamamatsu Photonics, Hamamatsu City, Shizuoka, Japan). NECTIN4, TROP2, and CEACAM5 expression was quantified using H-scores derived from automated image analysis in QuPath [13]. Briefly, cells were identified by QuPath’s built-in cell detection algorithm and further classified into tumor and non-tumor cells by a supervised machine learning–based object classifier. CD8^+^ T cells were detected by QuPath’s built-in positive cell detection algorithm and reported as cell density per unit area (cells/mm^2^). PD-L1 combined positive score (CPS), tumor proportion score (TPS), and immune cell (IC) score were manually assessed as described previously [14]. HER2 immunohistochemistry was manually scored according to the IHC scoring criteria of the ASCO/CAP guideline for HER2 testing in gastroesophageal adenocarcinomas [15]. A board-certified pathologist performed all manual scoring and plausibility checks of the automated analyses.

PD-L1 positivity was defined as CPS ≥ 10; TROP2 expression as high (2+ in >50% or 3+ in >10%) or low. For this exploratory analysis, HER2 IHC 2+ and 3+ cases were grouped as HER2 IHC-positive, reflecting moderate to strong protein expression rather than guideline-defined HER2 positivity, as no confirmatory ISH testing was performed. CEACAM5 expression was defined as high (2+/3+ > 50%), moderate (2+/3+ in 1%–50%), or negative. Optimal cut-off values for NECTIN4 H-score and CD8^+^ T cell density were determined using maximally selected rank statistics (survminer R package version 2023.06.0; NECTIN4: 34.14; CD8: 97.5).

### 2.3 Statistical Analysis

All statistical analyses were conducted using IBM SPSS Statistics software, version 29 (IBM Corp., Armonk, NY, USA). Continuous variables are reported as mean ± standard deviation (SD), depending on data distribution. Categorical and binary variables are presented as absolute frequencies and percentages. For histology-based analyses, case-level values were calculated by averaging the results obtained from the available TMA cores. Oncological outcomes, including OS and progression-free survival (PFS), were analyzed using the Kaplan–Meier method, with comparisons made using the log-rank test. Univariate and multivariate Cox proportional hazards regression analyses were performed to evaluate the effects of adjuvant chemotherapy and pT stage on survival. All statistical tests were two-tailed, and a *p*-value < 0.05 was considered statistically significant.

## 3 Results

### 3.1 Patient Characteristics and Postoperative Clinical Outcome

A total of 54 SCC cases were identified (25 pure, 29 partial SCC). After excluding 12 cases due to poor sample quality or incomplete data, the final TMA cohort included 42 patients (22 pure SCC, 20 partial SCC) (Fig. S1). Among the 54 patients, 51 underwent RC and three TURB only. The cohort comprised 27 men and 27 women with a mean age of 68.1 years (SD: 12.7). Most patients (54.9%) received an ileal conduit, followed by ureterocutaneostomy (19.6%) and Mainz pouch (17.6%).

Final histopathology among RC patients showed ≤pT1 in two (3.9%), pT2 in seven (13.7%), pT3 in 24 (47.1%), and pT4 in 18 patients (35.3%). Lymph node involvement was recorded as pN1 in seven (13.7%), pN2 in eleven (21.6%), and pN3 in three patients (5.9%). Five patients (9.8%) presented with distant metastases (M1).

None of the patients received neoadjuvant chemotherapy, whereas adjuvant chemotherapy was administered to twelve patients (23.5%). The mean OS was 14.5 months (SD 24.9), the mean PFS was 10.4 months (SD 21.1), and the mean follow-up duration was 11.9 months (SD 21.9) (Table 1).

**Table 1:** Overview of all 54 patients and the 42 cases represented in the tissue microarray (TMA), together with their clinical and pathological characteristics.

Patient Characteristics	Overall Patient Cohort (N = 54)	TMA Cohort (N = 42)
Age, years, mean (SD)	68.1 (12.7)	68.2 (11.7)
Sex, n^a^ (%)		
Men	27 (50)	22 (52.4)
Women	27 (50)	20 (47.6)
Pure SCC/partial SCC, n^a^/n^a^	25/29	22/20
RC/TURB only, n^a^/n^a^	51/3	40/2
Pathological T-stage at RC, n^a^ (%)		
≤pT1	2 (3.9)	2 (5)
pT2	7 (13.7)	6 (15)
pT3	24 (47.1)	17 (42.5)
pT4	18 (35.3)	15 (37.5)
Pathological N-stage at RC, n^a^ (%)		
pN0	25 (49)	17 (42.5)
pNx	5 (9.8)	5 (12.5)
pN1	7 (13.7)	4 (10)
pN2	11 (21.6)	11 (27.5)
pN3	3 (5.9)	3 (7.5)
Pathological M-stage at RC, n^a^ (%)		
M0	18 (35.3)	15 (37.5)
Mx	28 (54.9)	22 (55)
M1	5 (9.8)	3 (7.5)
Urinary diversion, n^a^ (%)		
Ileal conduit	28 (54.9)	23 (57.5)
Mainz pouch	9 (17.6)	6 (15)
Ureterocutaneostomy	10 (19.6)	8 (20)
Transverse colon conduit	1 (2)	1 (2.5)
Orthotopic neobladder	3 (5.9)	2 (5)
Surgical approach, n^a^ (%)		
Robotic-assisted RC	2 (3.9)	1 (2.5)
Open RC	49 (96.1)	39 (97.5)
Adjuvant chemotherapy, n^a^ (%)	12 (23.5)	10 (25)
Overall survival, months, mean (SD)	14.5 (24.9)	9.8 (11.2)
Progression-free survival, months, mean (SD)	10.4 (21.1)	6.8 (9.5)
Follow-up, months, mean (SD)	11.9 (21.9)	8 (10.3)

^a^Numbers reflect the number of patients (percentages); N, total patients; SD, standard deviation; SCC, squamous cell carcinoma; RC, radical cystectomy.

### 3.2 Immunohistochemistry

**Figure 1:**
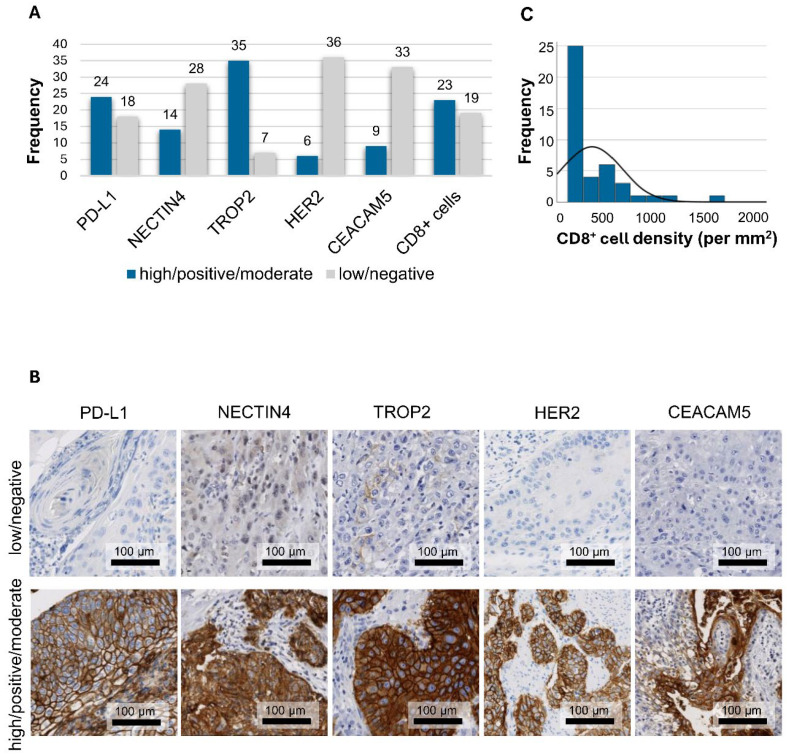
**Immunohistochemical targets and CD8^+^ T cell density.** (**A**) Expression in immunohistochemistry; immunohistochemical expression of each target was categorized as high/positive/moderate versus low/negative according to predefined criteria. (**B**) Representative immunohistochemical staining patterns of the investigated biomarkers in bladder cancer tissue samples. Representative photomicrographs illustrating low/negative expression (upper row) and high/positive/moderate expression (lower row) in patient samples. Scale bar: 100 μm. (**C**) CD8^+^ Cell Density Distribution; Distribution of CD8^+^ T cell density with overlaid density curve (black line).

PD-L1 expression showed a mean TC score of 17.7% (SD: 30.1), an IC score of 5.6% (SD: 6.7), and a CPS of 24.6 (SD: 31.4). Using a cutoff value of CPS ≥ 10, 24 samples (57.1%) were classified as PD-L1 positive. Positive NECTIN4 expression was detected in 14 cases (33.3%), with a mean H-score of 36.4 (SD: 35). High TROP2 expression was found in 35 samples (83.3%), with a mean H-score of 176.7 (SD: 70.6). HER2 expression was positive in six samples (14.3%). CEACAM5 showed moderate expression in nine samples (21.4%), with a mean H-score of 14.1 (SD: 24.7). CD8^+^ T cells were classified as positive in 23 samples (54.8%) (Fig. 1A,B, Table 2).

**Table 2:** Expression of therapeutic targets in the overall cohort and stratified by pure and partial SCC.

Target Expression	Partial SCC (n = 20)	Pure SCC (n = 22)	Total (n = 42)
PDL-1 (CPS ≥ 10), n^a^ (%)	12 (60)	12 (54.5)	24 (57.1)
NECTIN4 (positive), n^a^ (%)	6 (30)	8 (36.4)	14 (33.3)
TROP2 (high), n^a^ (%)	16 (80)	19 (86.4)	35 (83.3)
HER2 (positive), n^a^ (%)	4 (20)	2 (9.1)	6 (14.3)
CEACAM5 (moderate), n^a^ (%)	6 (30)	3 (13.6)	9 (21.4)
CD8 (high), n^a^ (%)	12 (60)	11 (50)	23 (54.8)

^a^Numbers reflect the number of patients (percentages).

The distribution of CD8^+^ T cell density was right-skewed, with the majority of cases showing low CD8^+^ cell densities and a few cases exhibiting markedly higher values. The median CD8^+^ cell density was 259.7 cells/mm^2^ (SD: 314.5) (Fig. 1C).

**Figure 2:**
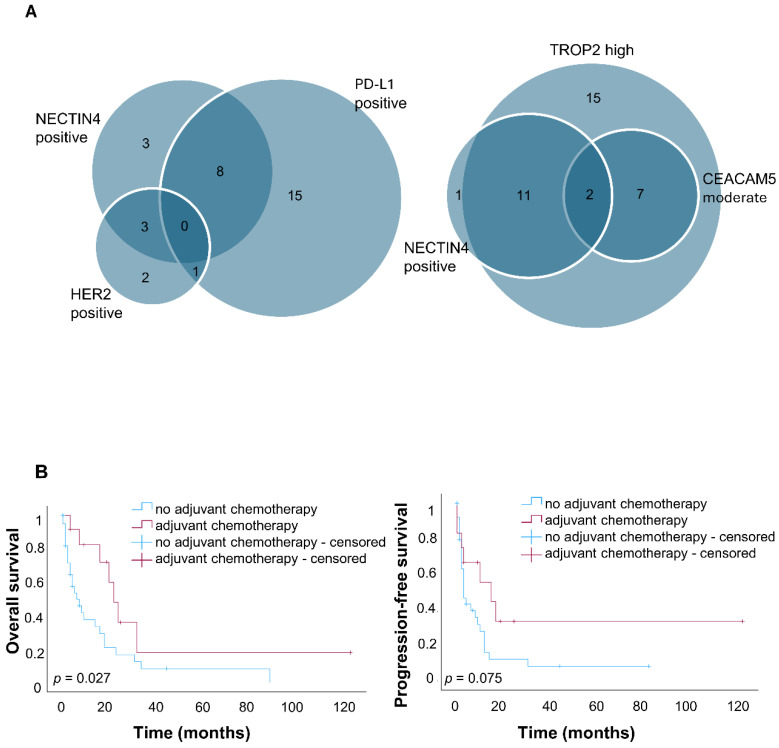
**Co-expression and impact of adjuvant chemotherapy on survival in bladder SCC.** (**A**) Overlapping Expression; Venn diagrams illustrating the overlapping expression of programmed death-ligand 1 (PD-L1), nectin cell adhesion molecule 4 (NECTIN4), and human epidermal growth factor receptor 2 (HER2) (left) and of trophoblast cell-surface antigen 2 (TROP2), NECTIN4, and carcinoembryonic antigen-related cell adhesion molecule 5 (CEACAM5) (right). Numbers indicate the number of cases in each category. (**B**) Adjuvant Chemotherapy and Survival Outcomes; Kaplan–Meier curves of overall survival (left) and progression-free survival (right) stratified by adjuvant chemotherapy.

Overlapping expression of NECTIN4 and PD-L1 was observed in eight cases. HER2 and NECTIN4 samples were simultaneously positive in three cases. Concurrent positivity for TROP2, CEACAM5, and NECTIN4 was detected in two cases, whereas co-expression of TROP2 and NECTIN4 was observed in eleven cases (Fig. 2A).

### 3.3 Prognostic Value of Adjuvant Chemotherapy and Therapeutic Targets

The administration of adjuvant chemotherapy was significantly associated with improved OS (*p* = 0.027), but not with PFS (*p* = 0.075) (Fig. 2B). In both the univariate and multivariate analyses, including T stage (non–muscle-invasive vs. muscle-invasive disease), the *p*-value for adjuvant chemotherapy remained statistically significant (Table S1).

**Figure 3:**
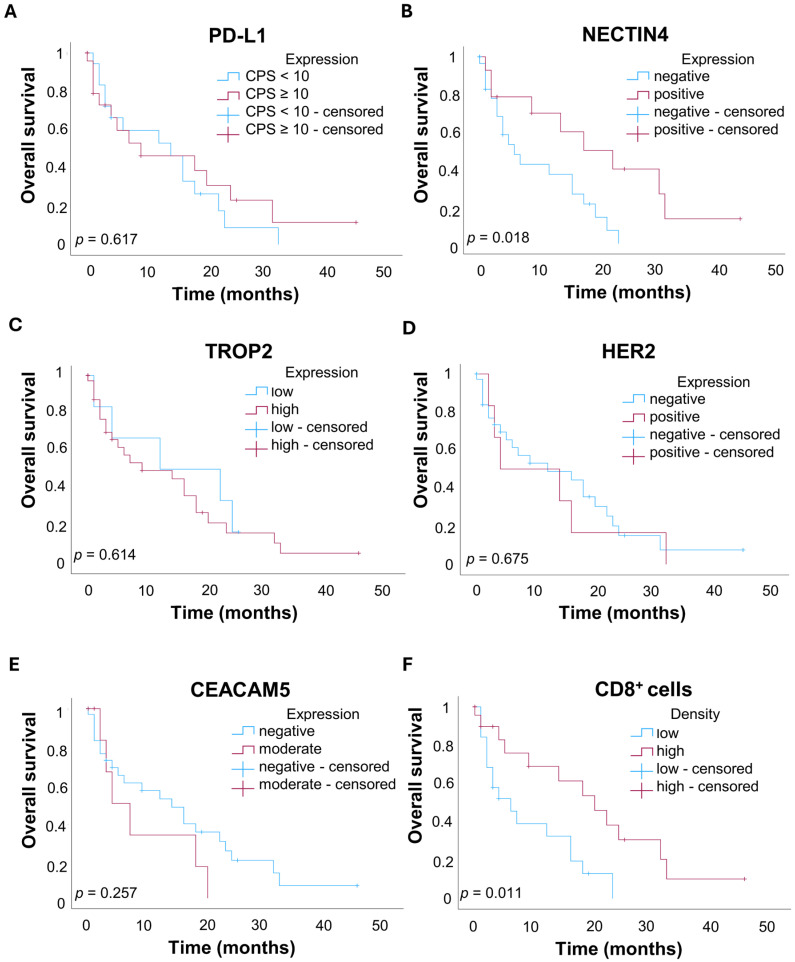
**Overall Survival of each Target.** Kaplan–Meier curves of overall survival for each target, with groups stratified by expression levels. (**A**) Programmed death-ligand 1 (PD-L1); (**B**) Nectin cell adhesion molecule 4 (NECTIN4); (**C**) Trophoblast cell-surface antigen 2 (TROP2); (**D**) Human epidermal growth factor receptor 2 (HER2); (E) carcinoembryonic antigen-related cell adhesion molecule 5 (CEACAM5); (F) CD8^+^ T cells.

High NECTIN4 expression was significantly associated with improved OS (*p* = 0.018), as was a high CD8^+^ T cell density (*p* = 0.011). No significant associations with OS were observed for any of the other targets analyzed, including PD-L1 (*p* = 0.617), TROP2 (*p* = 0.614), HER2 (*p* = 0.675), or CEACAM5 (*p* = 0.257) (Fig. 3). 

**Figure 4:**
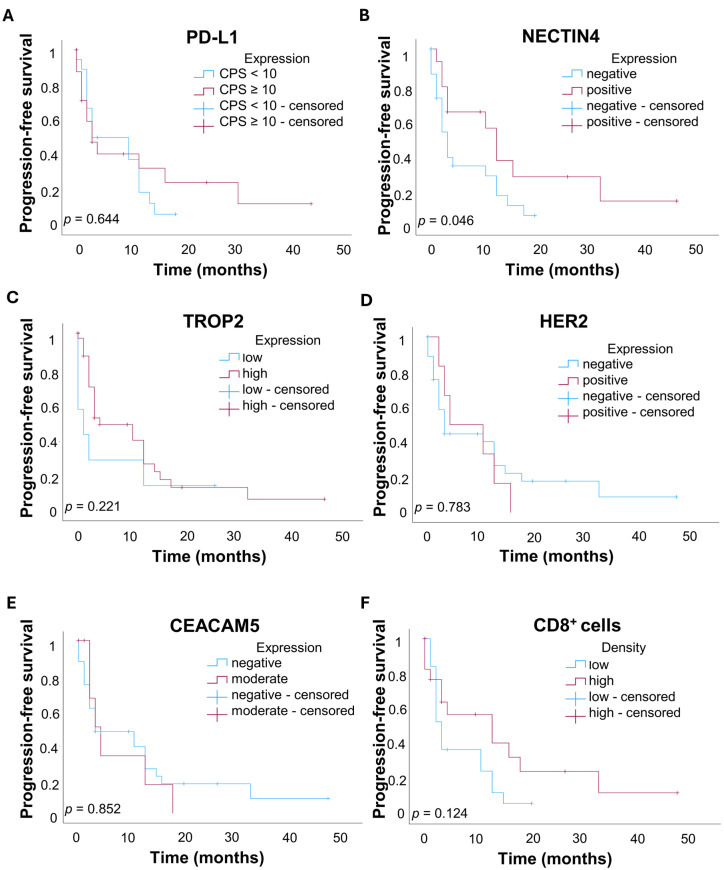
**Progression-free Survival of each Target.** Kaplan–Meier curves of progression-free survival for each target, with groups stratified by expression levels. (**A**) PD-L1; (**B**) NECTIN4; (**C**) TROP2; (**D**) HER2; (**E**) CEACAM5; (**F**) CD8^+^ T cells.

Regarding PFS, only NECTIN4 showed a significant association (*p* = 0.046) (Fig. 4).

## 4 Discussion

SCC of the bladder is one of the most common histologic subtypes and, due to its aggressive nature, remains a relevant therapeutic challenge in daily clinical practice. Our study provides valuable insights into the immunohistochemical expression of potential novel treatment targets, which could support personalized treatment approaches following failure of platinum-based chemotherapy.

Historically, PD-1/PD-L1 inhibitors were the first approved immune-related systemic therapies introduced for patients with advanced UC. However, early clinical trials comparing pembrolizumab to chemotherapy included only patients with advanced UC, thereby excluding those with SCC histopathology [16]. With the emergence of PD-1/PD-L1 inhibition, both the prognostic and, importantly, the predictive value of PD-L1 expression have been subjects of considerable debate [17]. In our cohort, PD-L1 expression—defined as a CPS ≥ 10—was evenly distributed, suggesting that PD-L1-targeted therapy might represent a potential treatment option. Nevertheless, both PD-L1 expression levels and the chosen testing methodology critically influence interpretability and clinical relevance. In a study by Morsch et al. [11], both pure and partially squamous differentiated SCC cases were analyzed, with a wide range of PD-L1 positivity depending on the antibody clone used (28–8, 22C3, SP142, or P263). PD-L1 was evaluated using the TC score, IC score, and CPS, with positivity ranging from 4.8% to 61.9% for IC and from 0% to 47.6% for TC, highlighting considerable heterogeneity in PD-L1 expression. Reis et al. reported high PD-L1 positivity rates in UC with variant histology, including SCC differentiation, with TC positivity ranging from 88–94% depending on the antibody clone, and therefore advocated for the inclusion of variant histologies in larger ICI trials [18].

As observed for PD-L1, NECTIN4 showed an even distribution of positive and negative expression. Consistent with our findings, Wucherpfennig et al. reported NECTIN4 expression with an H-score between 15 and 99 in 38% of cases and between 100 and 199 in 33% [19]. By contrast, they observed high NECTIN4 expression (H-score > 200) in 11% of patients, whereas none of the patients in our cohort reached an H-score above 200; the highest value recorded was 140. Interestingly, high NECTIN4 expression was associated with improved OS, whereas in the study by Rodler et al. NECTIN4 expression was not prognostic for OS [20]. Their analysis included only pure SCC cases (n = 61) and reported a median expression level of 150. These differences may reflect the inherent heterogeneity of NECTIN4 expression and the potential influence of partial SCC components on its prognostic value. 

Similar to the findings reported by Wucherpfennig et al., we observed high TROP2 positivity rates in our cohort (83.3% of cases), compared with 97% reported in their study [19]. TROP2 is a surface glycoprotein that is frequently highly expressed in a variety of epithelial malignancies [21]. However, high expression alone does not necessarily predict response to ADC therapies. Sacituzumab govitecan, an ADC targeting TROP2 and approved by the FDA in April 2021, did not demonstrate an improvement in OS or PFS compared with paclitaxel, docetaxel, or vinflunine in patients with UC progressing after platinum-based chemotherapy, as shown in the phase III trial by Powles et al. [10]. In light of these findings, the clinical development of sacituzumab govitecan in UC has been reconsidered, underscoring that high TROP2 expression alone may not translate into meaningful clinical benefit. Despite its limited impact on survival outcomes, this targeted therapy may retain clinical relevance for SCC of the bladder in the post-platinum-based setting, where alternative treatment options remain scarce.

In contrast to TROP2, HER2 expression was low in most cases. Importantly, HER2 expression, irrespective of its level, cannot necessarily be extrapolated across tumor entities concerning therapeutic efficacy. Recent studies in breast cancer have suggested that breast cancer patients with ultra-low HER2 expression can still benefit from the targeted therapy with trastuzumab deruxtecan, resulting in a longer PFS than chemotherapy after at least one line of endocrine-based therapy [22]. This concept of “ultra-low” HER2 expression has expanded the therapeutic landscape in breast cancer and highlights that even minimal receptor expression may be biologically and therapeutically relevant in certain tumor types. In a study by Hamilton et al., trastuzumab deruxtecan combined with nivolumab was evaluated in metastatic breast cancer and UC [23]. Among 30 UC patients with high HER2 expression, the response rate was 36.7%, while no responses were reported in four patients with low HER2; these rates were markedly lower than those reported in breast cancer, regardless of HER2 status, further emphasizing that HER2 biology and the predictive value of its expression may differ substantially between tumor entities such as breast cancer and UC.

In our study, CEACAM5 demonstrated only moderate expression in 21.4% of cases. In the study by Plage et al., CEACAM5 staining was largely absent in normal urothelium but was detected in approximately 30% of UC, comparable to our findings, and was particularly observed in a substantial proportion of pT2–pT4 tumors [24]. To the best of our knowledge, no studies to date have specifically investigated CEACAM5 expression in SCC of the bladder. Although several ADCs targeting CEACAM5 are currently under investigation, none have received regulatory approval to date [25].

We observed that high intratumoral density of CD8^+^ T cells was significantly associated with improved OS in this aggressive subtype of bladder cancer. CD8 is a surface marker of cytotoxic T lymphocytes, which are key effectors of the adaptive immune system. The role of CD8^+^ cells in cancer immunotherapy, as well as the development of chimeric antigen receptor (CAR)-T cell strategies, has been extensively investigated and has yielded substantial improvements in oncologic outcomes in various malignancies [26,27]. Consequently, our data indicate that bladder SCC exhibits a “hot” tumor immune microenvironment, which may render these tumors more susceptible to immune-based therapeutic interventions [28].

After assessing the expression of each target individually, we also analyzed the simultaneous expression of multiple targets. This approach is of particular interest, as combination therapies targeting two or more molecules may potentially enhance therapeutic efficacy. In our cohort, a relatively high proportion of cases (n = 11) demonstrated co-positivity for TROP2 and NECTIN4, suggesting that a combination of enfortumab vedotin and sacituzumab govitecan could represent a conceivable therapeutic strategy, provided that potential additive toxicities are carefully taken into account.

However, recent studies by Jindal et al. and Sternschuss et al. have reported limited efficacy of sacituzumab govitecan administered after enfortumab vedotin in metastatic UC, with objective response rates (ORR) of 18% and 11%, respectively [29,30]. Conversely, in metastatic UC patients treated with the combination of sacituzumab govitecan and enfortumab vedotin following platinum-based chemotherapy or immunotherapy, McGregor et al. observed an ORR of 70% (n = 23), although 78% of patients experienced grade ≥ 3 adverse events [31]. To date, there are no published data regarding such combination therapy in SCC, particularly in SCC cases exhibiting co-expression of both NECTIN4 and TROP2.

Among available adjuvant therapeutic strategies, chemotherapy remains one of the longest-established treatment options. In our cohort, more than 20% of patients received adjuvant chemotherapy, which was associated with improved OS. This aligns with findings of Koehne et al., who reported a notably low administration rate of only 11%, yet still observed a significant OS benefit [32], highlighting the continued relevance of chemotherapy in this aggressive tumor subtype.

This study has several limitations. A key limitation is its retrospective design and the relatively small cohort size of 42 patients, which particularly restricts the interpretability of subgroup analyses. Limited patient numbers frequently constrain studies focusing on bladder SCC and are therefore often grouped with other histologic subtypes. In contrast, the present study was intentionally dedicated exclusively to pure and partially squamous differentiated carcinomas to investigate multiple potential therapeutic targets despite the limited sample size. 

A further limitation is the lack of information on the exact extent of squamous differentiation, which was not reported in the pathology records. While IHC samples were selected based on the presence of squamous features, no conclusions can be drawn regarding cases with mixed urothelial and squamous differentiation.

As there are no established cutoffs for NECTIN4 H-Scores and CD8^+^ cell density, we relied on maximally selected rank statistics to define thresholds. This approach may reduce the reliability of the results and carries a risk of overfitting, potentially leading to false-positive survival associations.

## 5 Conclusions

In conclusion, our study identifies novel therapeutic targets in SCC, some of which already have corresponding targeted treatment options. Given the aggressive nature of this underrepresented histologic subtype and the current paucity of effective therapies, particularly in advanced stages, there is an urgent need for further therapeutic development and clinical evaluation.

## Data Availability

The data that support the findings of this study are available from the corresponding author, Lisa J. Frey, upon reasonable request.

## Supplementary Materials

The supplementary material is available online at https://www.techscience.com/doi/10.32604/or.2026.078954/s1. Figure S1: Patient Selection Flowchart; Flowchart of patient selection and sample inclusion for the pure and partial squamous cell carcinoma (SCC) cohort. 42 patients were included in the tissue microarray (TMA), comprising 22 pure SCC and 20 partial SCC cases. Table S1: Univariate and multivariate Cox proportional hazards regression analysis of adjuvant chemotherapy and pT stage.
