# Supplementary material for: Immunohistochemical Expression of Novel Therapeutic Targets in Squamous Cell Carcinoma of the Bladder
Source: Oncol Res. 2026 Jul 16;34(8):14. doi: 10.32604/or.2026.078954 (PMC13397361; doi:10.32604/or.2026.078954)
Supplement: Supplementary file 1 [file OncolRes-34-78954-s001.zip › TSP_OR_78954-s001.docx]

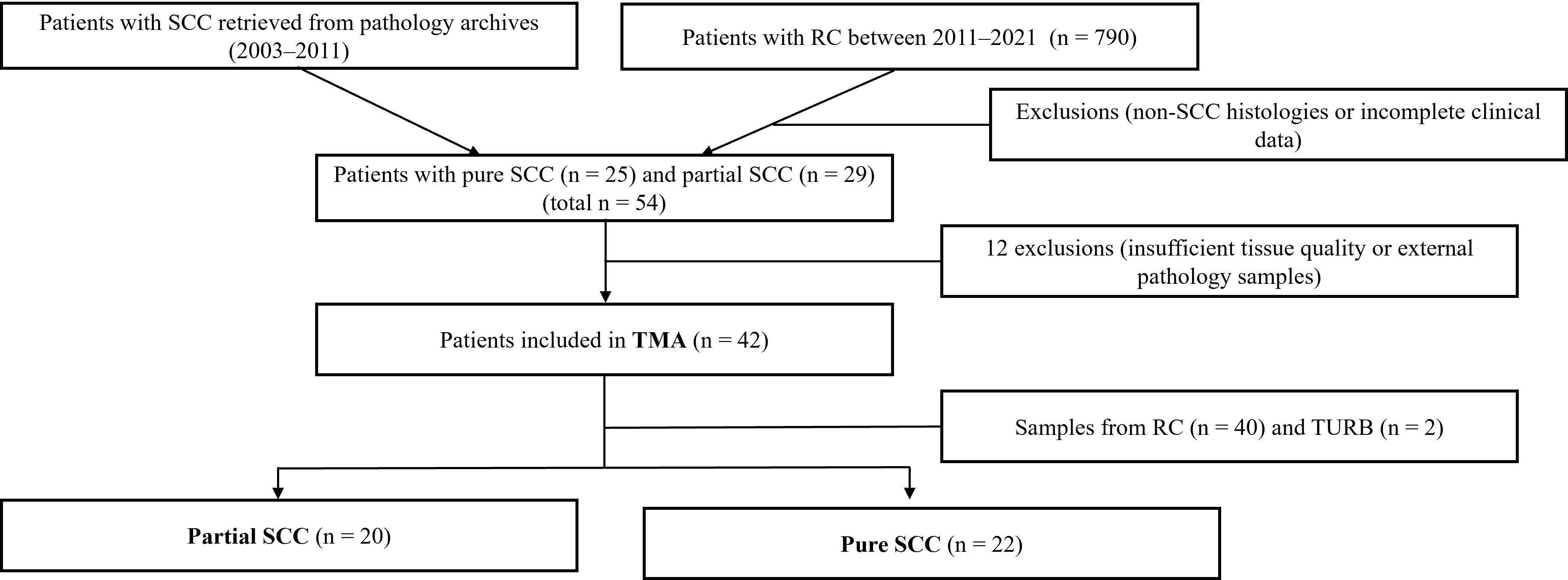


**Figure S1:** Patient Selection Flowchart; Flowchart of patient selection and sample inclusion for the pure and partial squamous cell carcinoma (SCC) cohort. 42 patients were included in the tissue microarray (TMA), comprising 22 pure SCC and 20 partial SCC cases.

**Table S1:** Univariate and multivariate Cox proportional hazards regression analysis of adjuvant chemotherapy and pT stage.

| **Variable** | **HR (95% CI), Univariate** | ***p*-Value** | **HR (95% CI), Multivariate** | ***p*-Value** |
| --- | --- | --- | --- | --- |
| <pT2 vs. ≥pT2 | 0.4 (0.093-1.718) | 0.218 | 0.179 (0.021-1.550) | 0.118 |
| Adjuvant chemotherapy | 0.404 (0.174-0.939) | **0.035*** | 0.412 (0.177-0.961) | **0.040*** |

HR, hazard ratio; CI, confidence interval; **p* < 0.05.
